# Fast eikonal phase retrieval for high-throughput beamlines

**DOI:** 10.1107/S1600577526004273

**Published:** 2026-06-19

**Authors:** Alessandro Mirone, Theresa Urban, Joseph Brunet, Claire L. Walsh, Peter D. Lee, Paul Tafforeau

**Affiliations:** a European Synchrotron Radiation Facility 71 Avenue des Martyrs F-38000Grenoble France; b Department of Mechanical Engineering University College London London United Kingdom; Paul Scherrer Institut, Switzerland

**Keywords:** phase retrieval, tomography, synchrotron imaging, propagation-based imaging, optics

## Abstract

A fast eikonal phase retrieval formulation is introduced that accelerates eikonal phase retrieval by more than two orders of magnitude while retaining controlled accuracy. The method is derived from a second-order asymptotic expansion in the propagation distance *L* and complemented by the leading Wentzel–Kramers–Brillouin (WKB) wave-optics correction, yielding an efficient iterative correction scheme preconditioned by fast Fourier transform (FFT)-diagonal, energy-dependent inverse operators (Paganin-type filters). To ensure robustness across practical experimental regimes, we combine two complementary solvers: (i) a local *O*(*L*^2^) closure that is accurate when eikonal shifts remain sub-pixel, and (ii) a non-local formulation for multi-pixel shifts, in which intensity is propagated through an explicit eikonal ray mapping using a mass-conserving bilinear redistribution on the detector grid, and detector residuals are transferred back to the object grid by the corresponding discrete adjoint (transpose) operator, implemented as bilinear interpolation, before applying an approximate FFT-diagonal preconditioner to accelerate convergence. The same framework supports polychromatic data through a compact spectral discretization, allowing energy-dependent transport and inversion while keeping the iteration efficient for graphics processing unit (GPU)/FFT. Overall, this unified approach enables accurate and computationally efficient phase retrieval across propagation conditions relevant to high-throughput propagation-based phase-contrast micro-tomography experiments.

## Abstract 2

Fast eikonal phase retrieval with complementary local (sub-pixel) and non-local (multi-pixel) solvers strongly suppresses nonlinear streak artefacts in long-distance propagation-based phase-contrast micro-tomography at high computational efficiency.

## 1. Introduction

Propagation-based phase-contrast micro-tomography (PPC-µCT) is one of the most widely used imaging modalities at synchrotron facilities, owing to its experimental simplicity, high photon efficiency and sensitivity across a broad range of applications. In its simplest implementation, provided that sufficient transverse coherence is available, PPC-µCT requires only moving the detector downstream with respect to a conventional absorption radiography set-up, which makes it particularly well suited to high-throughput imaging workflows.

The advent of fourth-generation synchrotron sources has increased beam brilliance while reducing the effective source size. As a consequence, longer sample-to-detector propagation distances can be exploited without incurring significant geometrical blur from the finite source size (circle of confusion). This enables operation in a propagation-enhanced near-field regime, where phase-contrast sensitivity is strongly increased and weakly refracting features can become visible above statistical noise. This regime is now routinely exploited on high-energy, high-throughput beamlines such as BM18 at the ESRF, notably in the context of hierarchical phase-contrast tomography (HiP-CT) (Walsh *et al.*, 2021 ▸).

However, the gain in sensitivity comes with important challenges. In realistic experiments on large and heterogeneous specimens, weakly refracting structures coexist with strongly absorbing regions and sharp interfaces, such as bone–air or bone–fluid boundaries. Biological tissues are organized hierarchically across multiple length scales, so weakly and strongly refracting structures naturally coexist within the same sample (Walsh *et al.*, 2021 ▸). At increased propagation distances, the intensity modulations generated by steep phase gradients at such interfaces are strongly amplified. Under these conditions, the linearized assumptions underlying standard single-distance phase retrieval (Paganin *et al.*, 2002 ▸) break down, leading to characteristic nonlinear artefacts (Mohan *et al.*, 2020 ▸; Mirone *et al.*, 2025*a* ▸; Urban *et al.*, 2025 ▸), including long-range streaks that severely degrade image quality and compromise downstream analysis.

A Fresnel wave-optics formulation (Mohan *et al.*, 2020 ▸) can, in principle, model near-field propagation even under strong refraction, but it requires an explicit pixel-wise representation of the complex wavefield. This introduces strict sampling constraints: when phase variations become too rapid to be sampled at the detector pitch, the discretized wavefield is no longer adequately band-limited and numerical Fresnel propagation becomes contaminated by aliasing unless very large oversampling factors are used. In propagation-enhanced PPC-µCT on heterogeneous specimens, sharp interfaces naturally produce such rapid phase variations, making brute-force Fresnel propagation quickly impractical in terms of memory footprint and computational cost.

By contrast, Paganin-type single-distance transport-of-intensity approaches, rooted in the transport-of-intensity equation introduced by Teague (1983 ▸) and specialized to homogeneous objects by Paganin *et al.* (2002 ▸), do not require an explicit pixel-wise phase map. Propagation is modelled through intensity transport under a linearized phase-gradient assumption, and the computation remains numerically stable even in situations where a fully sampled wavefield representation would be impractical. Their limitation is therefore primarily physical rather than numerical: once phase gradients and intensity dynamics become sufficiently strong, the linearization fails and nonlinear streak artefacts appear.

In our previous work we introduced the eikonal phase retrieval (EPR) algorithm, an iterative phase-retrieval framework based on the eikonal approximation (Mirone *et al.*, 2025*a* ▸). EPR was designed to go beyond the linearized transport-of-intensity description and to remain valid in the presence of strong phase gradients and large intensity dynamics, where direct Fresnel propagation becomes computationally impractical. Extensive experimental validation on challenging datasets, including strongly absorbing biological samples acquired on BM18, demonstrated that EPR suppresses or strongly reduces linearization artefacts and recovers fine structural details that are otherwise lost with standard linear approaches (Mohan *et al.*, 2020 ▸; Mirone *et al.*, 2025*a* ▸). In this sense, EPR established that a more faithful physical model of near-field propagation is essential in the propagation-enhanced phase-contrast regime to reach optimal sensitivity.

Despite these advantages, the first implementation of EPR suffered from a major practical limitation: computational cost. For large HiP-CT datasets, the full EPR workflow could require up to several tens of hours on multi-graphics processing unit (GPU) systems, whereas the experimental throughput of the beamline is typically of the order of one sample every few hours. This mismatch between reconstruction time and acquisition rate has so far hindered routine use.

Motivated by this limitation, several strategies were explored to reduce the computational burden. In particular, preliminary work investigated small convolutional neural networks to identify or correct the regions of the projections most responsible for nonlinear artefacts. Remarkably, these networks relied exclusively on local quantities, such as intensity values in a neighbourhood of each pixel and their planar derivatives, yet achieved promising results with relatively fast training (Admans, 2024 ▸). This observation suggests that, over a substantial fraction of the experimental regime of interest, the underlying physics is only weakly nonlinear and dominated by correction terms that admit a local differential representation.

This insight directly motivates the present work. We introduce a fast EPR formulation based on a reduced semi-analytic near-field model and an efficient fast Fourier transform (FFT)-preconditioned iterative inversion. In particular, we (i) retain the complete *O*(*L*^2^) content of a Wentzel–Kramers–Brillouin (WKB) (saddle-point) expansion of the Fresnel propagator, (ii) complement the local second-order closure with a non-local mapping-based solver robust to multi-pixel shifts, (iii) support polychromatic data through a compact spectral discretization, and (iv) achieve run times compatible with high-throughput reconstruction workflows while strongly suppressing nonlinear streak artefacts.

Section 2 summarizes the principle and algorithm at a level sufficient to interpret the results. Section 3 provides the methods used in the implementation, Section 4 gives the conclusion, and the supporting information collects the full derivations and additional implementation details.

## 2. Principle and algorithm overview

This section summarizes the forward model and inversion scheme used throughout the paper, introducing the notation needed to interpret the results. Full derivations and additional implementation details are provided in the *Methods* section[Sec sec4] and supporting information.

### 2.1. Forward model and WKB terminology

We describe near-field propagation using a WKB (Wentzel, 1926 ▸; Bender & Orszag, 1999 ▸) expansion of the Fresnel propagator. In this hierarchy, WKB0 corresponds to the leading eikonal (geometrical-optics) transport of intensity along rays, while WKB1 represents the first wave-optics correction (diffraction-pressure/quantum-potential term) (Madelung, 1927 ▸; Bohm, 1952*a* ▸; Bohm, 1952*b* ▸; Gureyev *et al.*, 1995 ▸). Accordingly, the detector intensity can be expressed as a linearized transport term plus higher-order corrections in the propagation distance *L*.

While higher-order terms can in principle be derived, their physical interpretation becomes progressively less transparent as one moves away from the near-field, single-valued-ray regime. Beyond the leading eikonal transport (WKB0), higher-order contributions increasingly encode wavefront physics that cannot be fully captured by a local, point-to-point WKB ray mapping. For this reason, we retain WKB0 and include only the leading WKB1 correction at *O*(*L*^2^) as a compact estimate of wave-optics effects within this near-field WKB framework.

### 2.2. Linear term and relation to Paganin

The *O*(*L*) term is the standard linearized transport-of-intensity description and, under the homogeneous-object assumption, its FFT-diagonal inverse reduces to the well known single-distance Paganin filter (Paganin *et al.*, 2002 ▸). In the iterative algorithm, this inverse is used as a *preconditioner* (Saad, 2003 ▸), *i.e.* an easily invertible approximate inverse that rescales the back-transported residual to accelerate convergence. We denote by 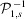 the FFT-diagonal approximate inverse of the linearized [*O*(*L*)] per-line operator (Paganin-type filter), and use it as a preconditioner.

### 2.3. Eikonal transverse shift and local versus non-local regime

Refraction induces a transverse ray displacement 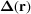 = 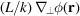, where 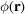 is the object-plane phase at *z* = 0 and *k* = 2π/λ. We characterize the regime by the shift-to-pixel ratio 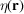 = 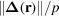, where *p* is the detector pixel size (or the internal step on an oversampled grid). When η < 1 over most of the field of view, the propagated intensity can be expanded locally in powers of *L*, leading to an efficient *O*(*L*^2^) differential forward model. When η > 1, the forward physics becomes effectively non-local: a detector pixel receives its dominant contribution from object-plane locations displaced by one or more pixels. In that regime, we retain the explicit WKB0 ray mapping and evaluate the forward operator numerically as a conservative bilinear redistribution on the detector grid. The detector residual is then transferred back to the object grid with the corresponding adjoint (transpose) operator, implemented as bilinear interpolation at the mapped coordinates, after which the preconditioner 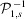 is applied to accelerate convergence.

### 2.4. Polychromatic data

Polychromatic measurements are modelled as an incoherent sum of *N*_*s*_ spectral components. All per-line quantities (phase/absorption parameters and shifts) are treated energy-dependently while keeping the iteration FFT efficient. If the effective spectrum is spatially uniform, the model reduces to a single-spectrum description; if needed, it can also accommodate slow spatial variations of the effective spectrum across the detector (see *Methods*[Sec sec4] and supporting information).

**Figure 1:**
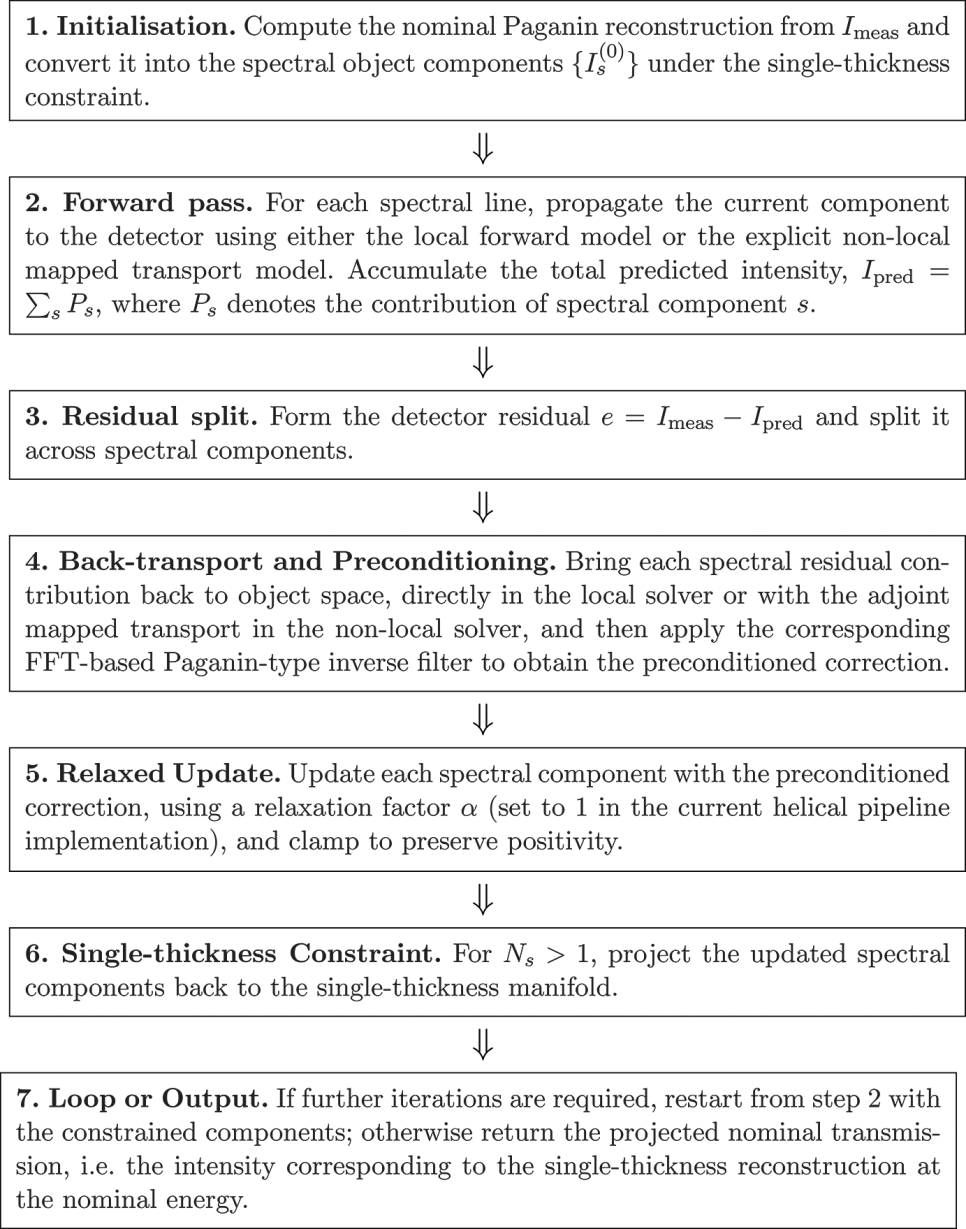
Structured workflow of one fast-EPR iteration. The same iteration skeleton is used in the local and non-local solvers; only the forward/backward transport pair changes between the two regimes. Optional implementation details such as warm-up scheduling, edge blending/apodization, post-retrieval unsharp masking, and frozen shifts are omitted here for clarity.

Fig. 1 ▸ summarizes the practical phase-retrieval workflow used in the local and non-local solvers.

## 3. Results

### 3.1. Sheep-head HiP-CT dataset and region of interest

Here we apply the local and non-local methods to the sheep-head HiP-CT dataset (Urban *et al.*, 2025 ▸), previously used to introduce and validate the original EPR method. The experiment follows the HiP-CT protocol, where the specimen is mounted in ethanol and acquired at long propagation distance to maximize propagation-based contrast on weakly absorbing soft tissues. This dataset is particularly challenging because the slice contains, simultaneously, (i) low-contrast anatomical structures (soft tissues) and (ii) very strong phase gradients and absorption variations associated with bones and bone interfaces, which generate pronounced propagation-induced nonlinear effects and long-range artefacts.

**Figure 2:**
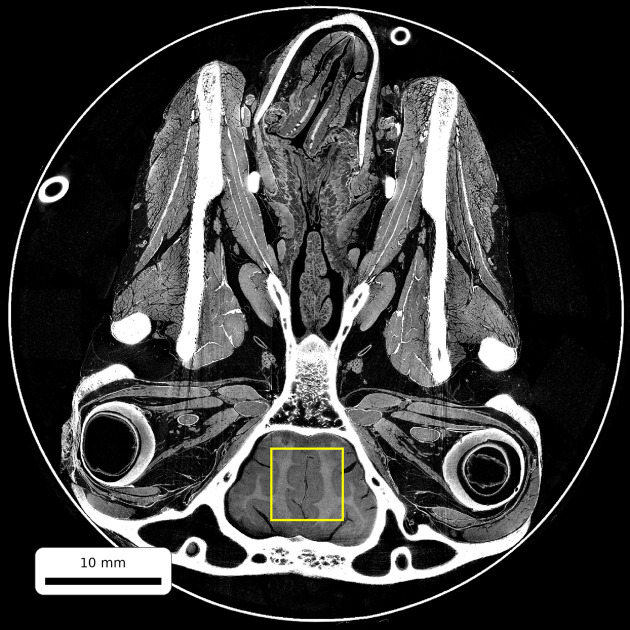
Full reconstructed slice of the sheep-head HiP-CT dataset (same specimen and acquisition context as in the original EPR paper). The yellow rectangle marks the region of interest (ROI) used for the detailed comparisons in Figs. 3 ▸–9 ▸. Among the multiple zoomed regions discussed in the original EPR work, the ROI chosen here corresponds to the brain region (inset *A* in Fig. 3 of the original EPR paper), where soft-tissue contrast coexists with strong nearby gradients induced by adjacent bone structures. Scale bar: 10 mm.

The region of interest (ROI) depicted in Fig. 2 ▸ was selected to emphasize the regime most relevant to this work: the simultaneous presence of weak features and strong gradients, for which a purely linearized, single-distance forward model is known to produce characteristic artefacts. In particular, this brain-region ROI is representative because it contains fine anatomical texture in soft tissues, while remaining influenced by high-gradient structures (bones and interfaces) that drive the strongest propagation nonlinearities.

### 3.2. Effect of the second-order (*L*^2^) forward model and of polychromatic modelling

**Figure 3:**
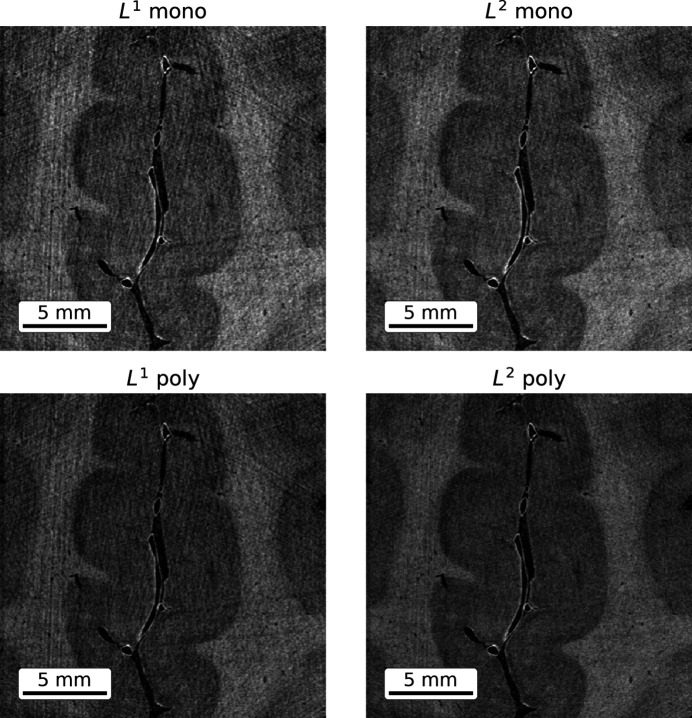
ROI comparison (brain region) for the four forward models. Top row: monochromatic models; bottom row: polychromatic models. Left column: first-order model *L*^1^; right column: second-order model *L*^2^. [Here *L*^1^ mono is equivalent to the Paganin single-distance forward model in the homogeneous-object setting (Paganin *et al.*, 2002 ▸).] Scale bars: 5 mm in each panel.

We compare four reconstructions of the same ROI, arranged in the 2 × 2 grid of Fig. 3 ▸. The top row reports the monochromatic approximation (‘mono’), while the bottom row reports the polychromatic model (‘poly’). The left column corresponds to the first-order propagation model *L*^1^, *i.e.* the forward (eikonal/WKB0 transport) truncated at *O*(*L*), whereas the right column corresponds to the second-order model *L*^2^, *i.e.* the forward truncated at *O*(*L*^2^) and including the additional diffraction-pressure (WKB1) contribution that enters the intensity at second order. [In the homogeneous-object setting, *L*^1^ mono is equivalent to the standard Paganin phase-retrieval single-distance model (Paganin *et al.*, 2002 ▸).]

A first observation from Fig. 3 ▸ is that the polychromatic modelling is consistently beneficial: the *L*^1^ poly reconstruction is slightly improved with respect to *L*^1^ mono, and the same trend is observed between *L*^2^ poly and *L*^2^ mono. This is expected, as the effective spectrum alters the mapping between absorption and phase shift and therefore the quantitative balance between attenuation and refraction in the forward model.

The dominant improvement, however, is obtained by moving from *L*^1^ to *L*^2^. Including the full second-order content (quadratic eikonal WKB0 transport plus the WKB1 diffraction-pressure correction) reduces the residual nonlinear propagation artefacts that persist under the first-order truncation, and provides a visibly cleaner rendering of soft-tissue contrast in the presence of nearby strong gradients. In practice, the *L*^2^ models better suppress the characteristic propagation-induced distortions that remain when only the first-order transport term is retained.

**Figure 4:**
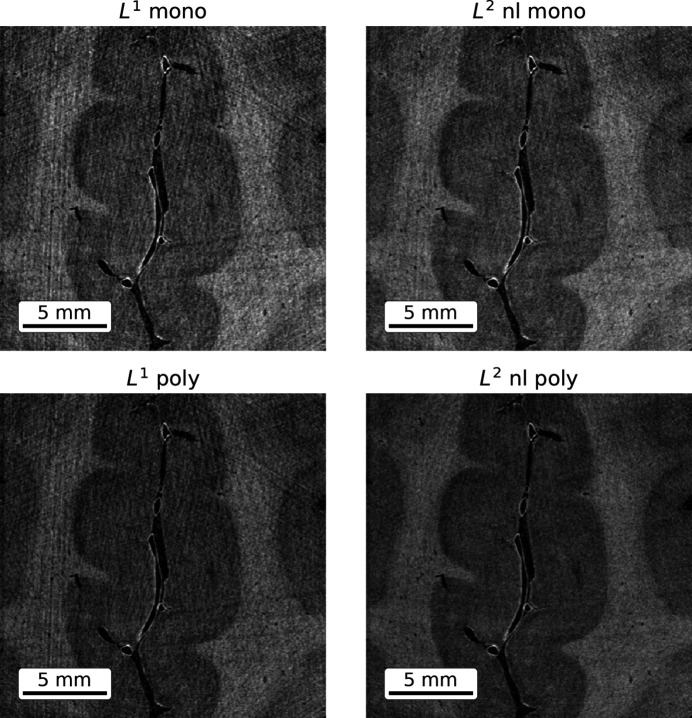
Non-local: ROI comparison (brain region) for the four forward models. Top row: monochromatic models; bottom row: polychromatic models. Left column: first-order model *L*^1^; right column: second-order non-local model *L*^2^. [Here *L*^1^ mono is equivalent to the Paganin single-distance forward model in the homogeneous-object setting (Paganin *et al.*, 2002 ▸).] Scale bars: 5 mm in each panel.

In Fig. 4 ▸ the same comparison is repeated with the non-local solver, which remains stable and accurate when the estimated shift distribution extends beyond one pixel.

**Figure 5:**
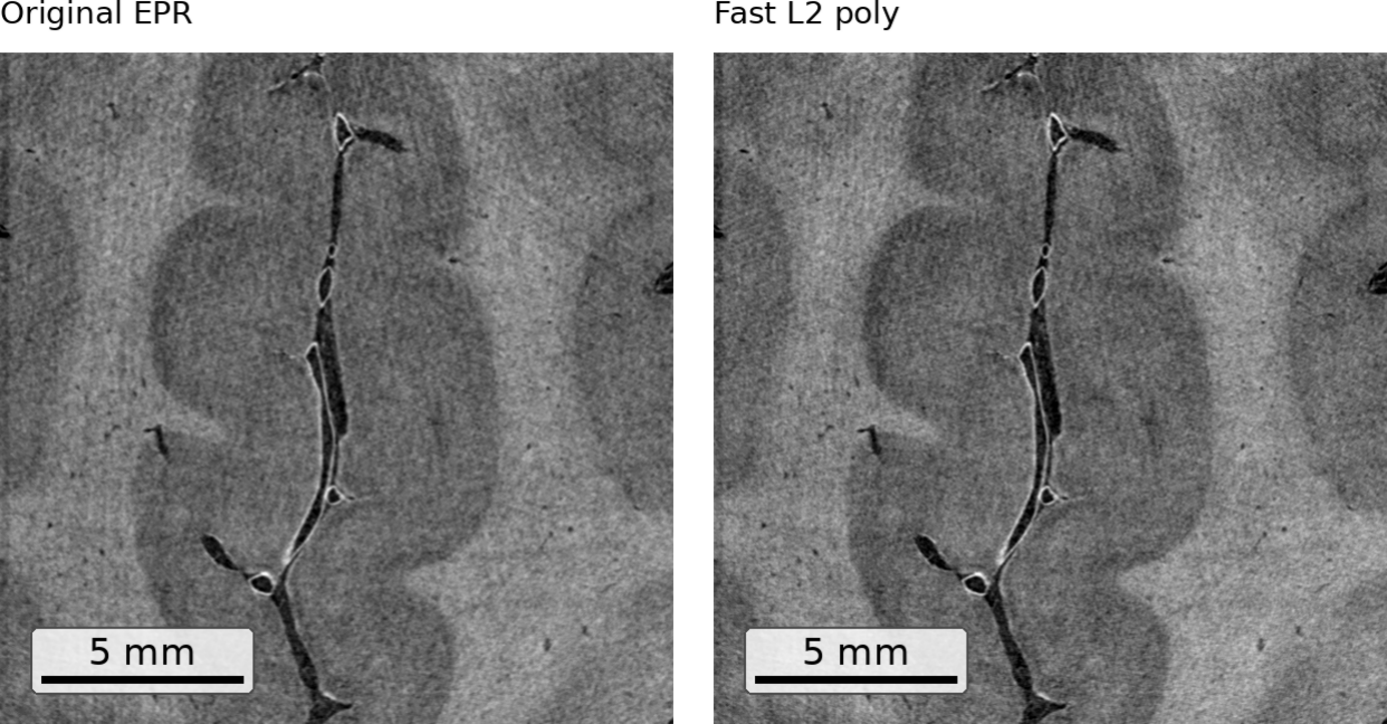
Direct comparison in the reconstructed tomographic domain on the same ROI between the original ray-mapping EPR approach (left) and the proposed accelerated FFT-based *L*^2^ polychromatic reconstruction (right). Both panels are shown with the same ROI crop, grayscale window and scale bar.

**Figure 6:**
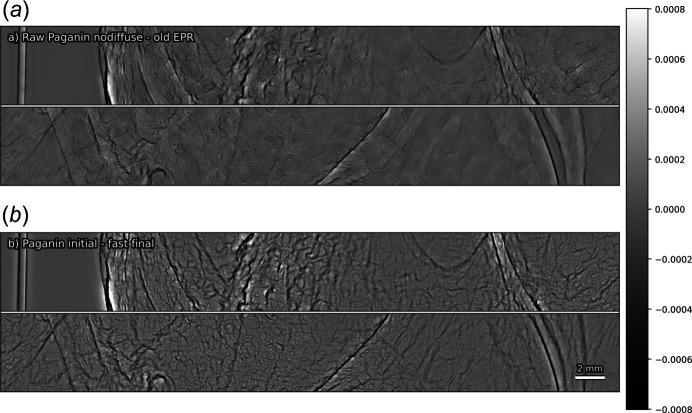
Direct comparison in the projection domain for a representative retrieved radiograph. Panel (*a*) shows the difference between a simple single-distance Paganin retrieval applied directly to the raw radiograph and the original EPR retrieved projection. Panel (*b*) shows the difference between the initial Paganin estimate used by the fast solver and the final fast retrieved projection. In each panel, the wide projection-difference image is split into left and right halves and these halves are stacked vertically for display, with a horizontal divider marking the join. Both panels are displayed with the same crop and the same grayscale range.

**Figure 7:**
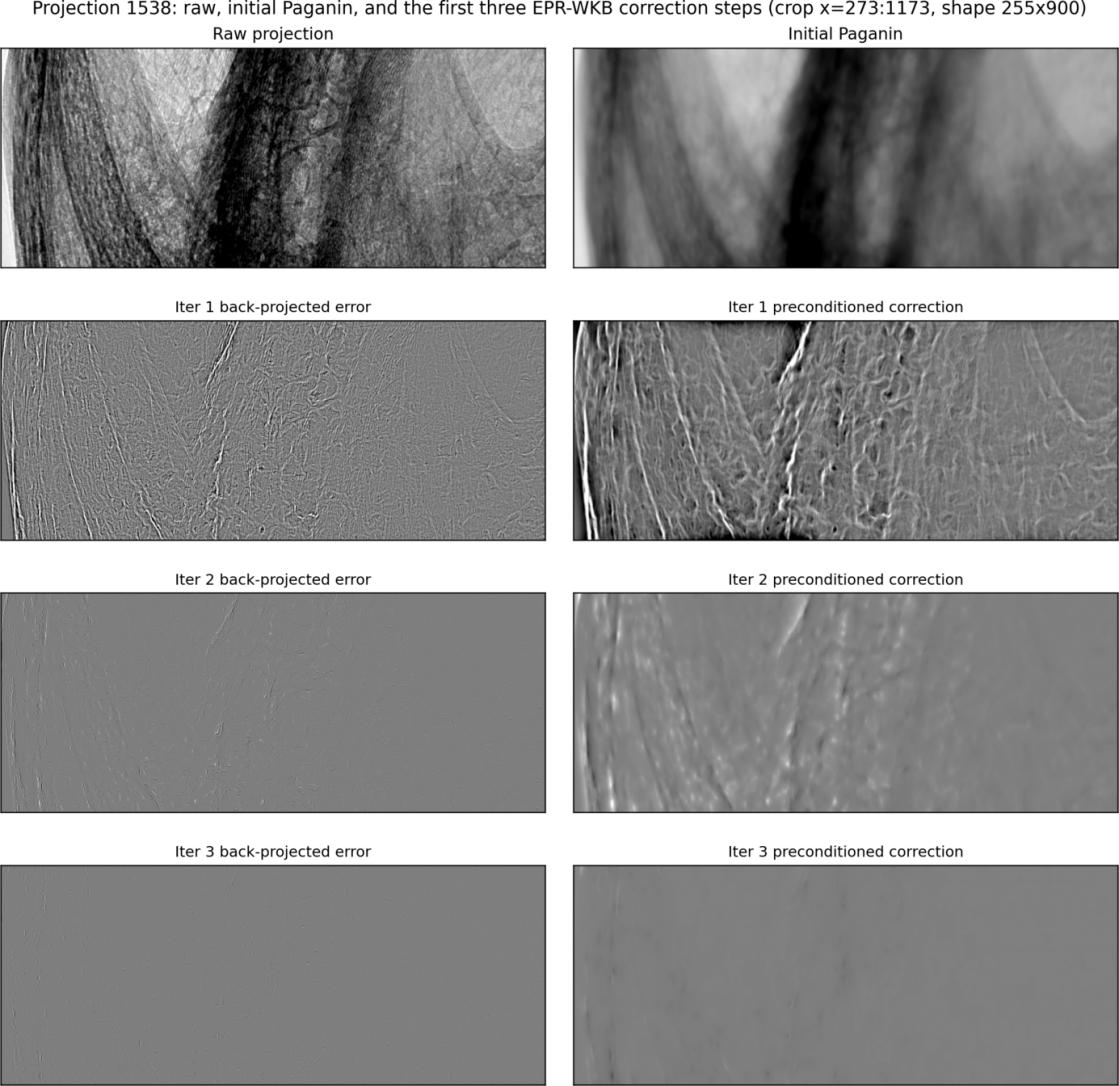
Evolution of the fast non-local phase retrieval in the projection domain for the same representative radiograph. The first row shows the raw projection and the initial Paganin estimate. The following rows show the back-projected residual and the preconditioned correction after iterations 1, 2 and 3. For each quantity type, the same grayscale range is used across iterations, making the rapid decrease of the correction amplitudes directly visible.

A direct comparison in the reconstructed tomographic domain between the proposed accelerated FFT-based formulation and the original ray-mapping EPR approach is provided in Fig. 5 ▸. On this ROI, the two reconstructions remain visually very close when shown with the same crop and grayscale window, supporting the conclusion that the computational speed-up does not come at a visible loss of image quality in this regime. The corresponding comparison in the projection domain is shown in Fig. 6 ▸, while Fig. 7 ▸ illustrates how the fast solver modifies a representative projection over the first three iterations. Although the retrieved projections themselves remain close, the difference images with respect to the corresponding Paganin references show that the fast reconstruction follows the fine sample structures more naturally. This is a strong indication that the rapid convergence of the new algorithm allows it to approach the converged nonlinear solution more effectively, whereas the original EPR implementation relied on a much more laborious multiscale optimization, based on successive conjugate-gradient stages carried out at different scales and with truncation indices chosen as a compromise between image quality and execution time.

**Figure 8:**
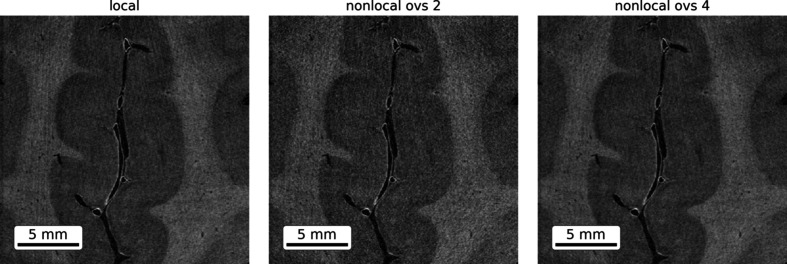
Local versus non-local phase retrieval on the same ROI. Left: local (*L*^2^) solver (oversampling factor 2). Centre: non-local solver with oversampling factor 2, showing residual high-frequency noise. Right: non-local solver with oversampling factor 4, where this noise is strongly reduced. Scale bars: 5 mm in each panel.

All reconstructions reported in this section were computed on oversampled grids. Unless stated otherwise, the local solver uses an oversampling factor of two (*i.e.* a pixel size reduced by a factor of two), while the non-local solver uses an oversampling factor of four. Oversampling improves numerical robustness in two distinct ways. First, it reduces discretization error in the transverse derivatives (gradients, Laplacians and divergences) used by the local *L*^2^ forward model. Second, in the non-local solver, it reduces quantization effects in the discrete ray-map transport implemented by bilinear redistribution on the detector grid and the corresponding discrete adjoint (transpose) interpolation back to the object grid, which can otherwise manifest as residual high-frequency texture when the displacement field spans a significant fraction of a pixel. This effect is illustrated in Fig. 8 ▸, which compares the local solver (left) with the non-local solver at oversampling factors of two (centre) and four (right). The residual high-frequency pattern visible in the non-local reconstruction at oversampling of 2 is strongly reduced when increasing the oversampling to 4.

### 3.3. Fast convergence of the *L*^2^ polychromatic inversion

**Figure 9:**
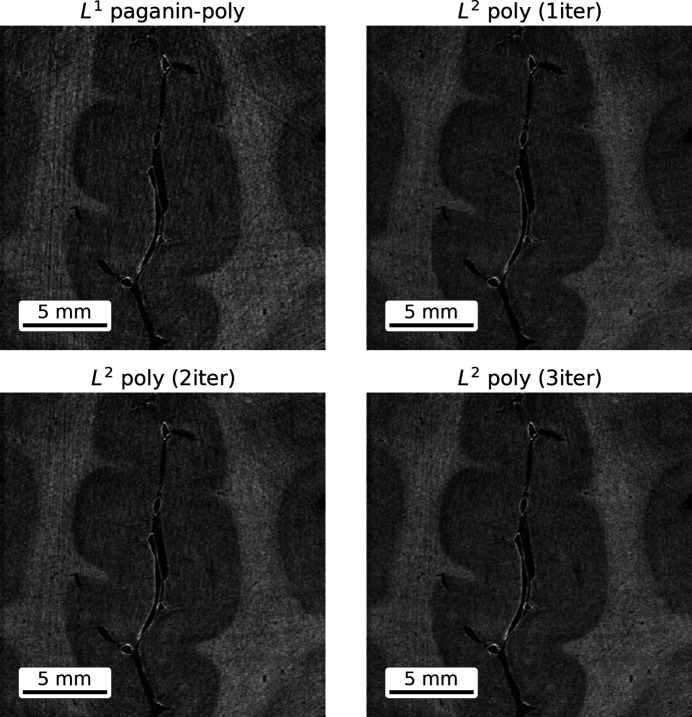
Convergence in the polychromatic case. A large improvement is already achieved when switching from *L*^1^ poly to *L*^2^ poly with a single iteration. Subsequent iterations produce only minor (often barely visible) refinements, indicating rapid convergence of the *L*^2^ polychromatic inversion in this ROI. Scale bars: 5 mm in each panel.

Fig. 9 ▸ focuses on convergence in the polychromatic setting. For reference, the top-left panel reports the first-order solution (*L*^1^ poly). The remaining panels show the second-order reconstruction (*L*^2^ poly) after one, two, and three iterations. This makes it possible to assess both the improvement obtained when enabling the full *O*(*L*^2^) forward physics and the marginal effect of additional iterations once the *L*^2^ model is used.

The key result in Fig. 9 ▸ is that the benefit of the second-order model appears immediately: going from *L*^1^ poly to *L*^2^ poly (one iteration) already yields most of the visible improvement. Increasing the iteration count beyond the first *L*^2^ update leads to changes that are comparatively subtle, suggesting that the algorithm converges very rapidly once the correct second-order physics is enabled in the forward model.

### 3.4. Effect of the diffraction-pressure term

Across the propagation distances and sample regimes explored in this work, the diffraction-pressure (WKB1) contribution is consistently negligible compared with the dominant WKB0 (eikonal transport) terms. We nevertheless include it in the forward model as the leading wave-optics correction in the same second-order expansion framework, and because it may become relevant in regimes closer to the boundary of validity of near-field approximations (*e.g.* at lower energy and/or higher spatial resolution), where diffraction effects can no longer be safely ignored.

### 3.5. Robustness stress test beyond the sub-pixel regime: bamboo sample

**Figure 10:**
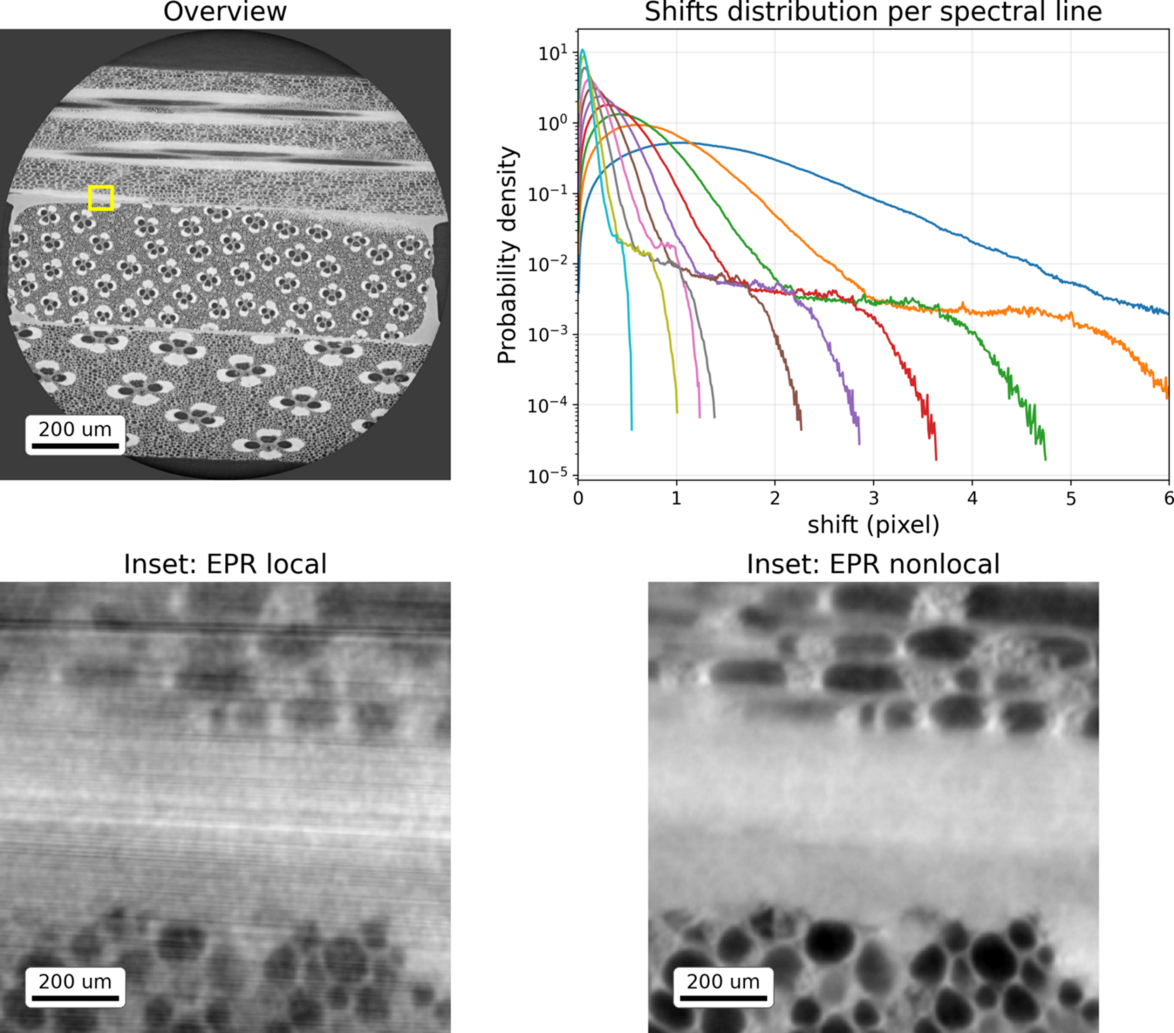
Zoom on a bamboo region acquired at 1.12 µm voxel size, 71 keV average beam energy, and 250 mm propagation distance. Top left: overview. Top right: distribution of estimated WKB0 transverse shifts (in detector-pixel units) for each sampling energy (spectral line) on a representative radiograph. Bottom left: local solver after two iterations (diverging). Bottom right: non-local solver with 10 spectral lines, 5 iterations. Scale bars: 200 µm in the spatial-image panels.

Fig. 10 ▸ reports a challenging bamboo sample acquired at 1.12 µm voxel size, with an average beam energy of 71 keV and a propagation distance of 250 mm. This long propagation distance was intentionally chosen, given the pixel size, as a stress test for the sub-pixel assumptions underlying the local solver.

The top-right panel of Fig. 10 ▸ shows the distribution of estimated WKB0 transverse shifts (in detector-pixel units) for each sampling energy (spectral line) on a representative radiograph. A significant fraction of the field of view exhibits shifts larger than one pixel, placing this dataset outside the regime where a purely local *O*(*L*^2^) closure is expected to remain accurate and stable. In this regime, the local solver fails (it degrades already at the first iteration and subsequently diverges), whereas the non-local solver remains stable. The bottom row of Fig. 10 ▸ compares the local EPR result after two iterations (left) with the non-local result (right), obtained using ten spectral lines, five iterations, and a relaxation factor of 0.4. The divergence appears first where the strongest gradients are, along the long longitudinal features. The purpose of this bamboo dataset is to validate robustness when refraction-induced shifts exceed the detector pixel size. In this strong-shift regime, the mapping-based non-local solver remains stable, while the local *O*(*L*^2^) closure breaks down. In this particular stress-test we are already beyond the near-field limit where a single-valued eikonal, point-to-point mapping is justified. Extending the model beyond the eikonal setting is outside the scope of the present work and will be addressed in future developments.

### 3.6. Computational performance

We report indicative run times (see Table 1 ▸) on a representative dataset comprising 8000 radiographs of size 256 × 3104 pixels (about 6.4 × 10^9^ pixels in total). All timings include radiograph reading (I/O) and the phase-retrieval computation, but exclude tomographic backprojection.

The benchmarks were obtained on a shared-memory server providing 96 CPU cores (AMD EPYC 75F3 class) and one NVIDIA A40 GPU. The examples shown in this section can be reproduced by installing and running the NightRail workflow (Mirone *et al.*, 2025*b* ▸), as detailed in the supporting information.

#### 3.6.1. Baseline (*L*^1^ mono, Paganin)

The first-order monochromatic model (*L*^1^ mono), equivalent to the standard Paganin single-distance retrieval under the homogeneous-object assumption, requires approximately 45 s on the 96-core CPU node.

#### 3.6.2. Second order (*L*^2^ mono)

With the second-order model (*L*^2^ mono), the run time increases due to the additional planar-derivative evaluations required by the *O*(*L*^2^) terms. For one iteration, the run time is approximately 1.9 min on CPUs and 1.1 min on one GPU; for two iterations it is about 2.7 min (CPU) and 1.23 min (GPU).

#### 3.6.3. Second order (*L*^2^ poly)

Using a five-line spectral discretization (polychromatic model) increases the computational cost. For one iteration, we measure 6.7 min on CPUs and 1.47 min on one GPU; for two iterations, 11.3 min (CPU) and 1.9 min (GPU).

#### 3.6.4. Non-local solver (multi-pixel shifts)

In the strong-shift regime, the non-local solver is more robust but requires additional resampling work. With five spectral lines, one iteration requires 2 min on one GPU for an oversampling factor of two, and about 8 min for an oversampling factor of four.

#### 3.6.5. Comparison with the original EPR implementation

For the same dataset size and geometry the original EPR benchmark reported on the order of one and a half days on 12 NVIDIA A40 GPUs for a dataset corresponding to approximately ∼30 comparable volumes and polychromatic treatment; this yields an effective cost of ∼72 min per volume on 12 GPUs, essentially all consumed by the phase retrieval process. Compared with the original EPR benchmark reported by Mirone *et al.* (2025*a* ▸), the present implementation reduces the phase-retrieval wall time per comparable volume by a factor of 580 when normalized to the same GPU resources (about 2.8 orders of magnitude) for one iteration with five-line spectral discretization, enabling routine use in high-throughput workflows.

## 4. Methods

In this section we summarize the forward operators and the inversion scheme used in the implementation, and we provide the final expressions required to reproduce the algorithm. The full saddle-point/WKB derivation of the local *O*(*L*^2^) intensity expansion is provided in the supporting information.

### 4.1. Local forward model summary: *O*(*L*^2^) closure with WKB terminology

We work with transverse coordinates 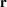 = (*x*, *y*) in the object plane (*z* = 0) and 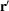 = 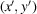 in the detector plane (*z* = *L*). The scalar complex field is written as 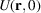 = 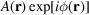, with intensity 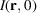 = 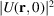 = 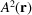. We denote by 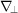 derivatives with respect to the transverse coordinates, and *k* = 2π/λ is the wavenumber.

We introduce ɛ ≃ *L*/*k* and use a WKB expansion of the Fresnel propagator. In this hierarchy, WKB0 denotes the leading eikonal transport (ray map/intensity transport), while WKB1 denotes the first wave-optics correction (diffraction-pressure/quantum-potential term). The resulting local intensity expansion reads, using Einstein summation convention for repeated indices, 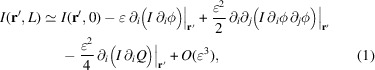
where 
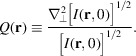
The complete derivation is given in the supporting information. The last term in equation (1), involving *Q*, corresponds to the previously mentioned diffraction-pressure contribution.

While higher-order terms can in principle be derived, beyond *O*(*L*^2^) they increasingly encode wavefront/interference physics that cannot be represented fully within a local, single-valued WKB mapping. For this reason, we truncate at *O*(*L*^2^) (WKB0 plus the leading WKB1 contribution) in the present work.

### 4.2. Non-local (multi-pixel) forward model: explicit WKB0 transport and discrete adjoint

Throughout the *Methods* section[Sec sec4], we denote by **Δ**(**r**) the physical eikonal-induced transverse shift, while *p* denotes the detector pixel size.

#### 4.2.1. Motivation and regime

The local second-order closure equation (1) is obtained by Taylor-expanding WKB0 transport in powers of ɛ = *L*/*k*. This truncation is accurate when the associated eikonal ray shifts are typically sub-pixel. When shifts become comparable with, or larger than, the pixel size, the Taylor closure breaks down. To preserve robustness in this multi-pixel regime, we therefore replace the local Taylor closure by an explicit discrete realization of the WKB0 mapping itself.

#### 4.2.2. Per-line eikonal mapping and shift field

The WKB0 stationary-point condition defines the ray mapping, 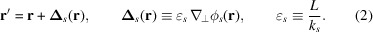
In practice we work in pixel units 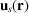 = 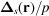, where *p* is the detector pixel size (or the internal pixel size on the oversampled grid).

#### 4.2.3. Non-local forward operator as explicit WKB0 ray mapping (no Taylor expansion)

The non-local forward model retains WKB0 forward mapping without expanding the delta,
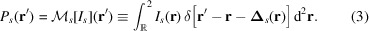
For a fixed shift field 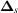, 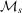 is linear in *I*_*s*_ and represents a mass-conserving transport. When 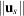

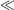
 1 pixel, the Taylor expansion of equation (3) recovers the local eikonal series [and, when complemented by the second-order corrections, equation (1)].

#### 4.2.4. Discrete implementation: conservative bilinear redistribution on the detector grid

On a Cartesian detector grid, equation (3) is evaluated by a conservative resampling: each source pixel at integer coordinate (*x*, *y*) contributes its mass *I*_*s*_(*x*, *y*) to the mapped location 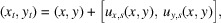
which generally lies between pixels. The mass is distributed to the four surrounding detector pixels using bilinear weights. Denoting 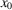 = 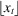, 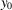 = 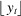, *d*_*x*_ = *x*_*t*_ − *x*_0_, *d*_*y*_ = *y*_*t*_ − *y*_0_, the weights are 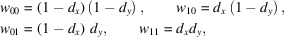
and the prediction is accumulated as 
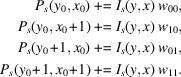


#### 4.2.5. Adjoint (transpose) operator: bilinear interpolation of the detector residual

With this definition, the back-transport of a detector residual is performed with the exact adjoint (transpose) of the discrete bilinear redistribution operator. In practice, this adjoint action is numerically equivalent to bilinear interpolation of the detector residual at the mapped location (*x*_*t*_, *y*_*t*_),
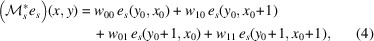
which is numerically equivalent to bilinear interpolation of *e*_*s*_ at (*x*_*t*_, *y*_*t*_), but written in this explicit adjoint form to make the transpose relationship clear.

### 4.3. Polychromatic framework: spectral discretization (local and non-local)

#### 4.3.1. Polychromatic measurement as an incoherent spectral sum

We discretize the spectrum into *N*_*s*_ effective spectral points (‘lines’) indexed by *s* = 1,…, *N*_*s*_. The detector intensity is modelled as an incoherent sum of the propagated intensities of the spectral components, 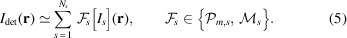
In the local formulation we take 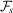 = 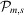, *i.e.* the Taylor/WKB closure truncated at order *m* ∈ {1, 2}. In the non-local formulation we take 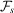 = 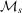, *i.e.* the explicit WKB0 transport operator equation (3).

#### 4.3.2. Single-material phase–absorption relation (per spectral line)

For a single-material (homogeneous) object with refractive index *n* = 1 − δ + *i*β, both phase and attenuation are proportional to the projected thickness, and eliminating thickness yields 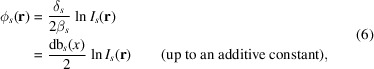
following the standard homogeneous-object relation used in single-distance phase retrieval and omni-microscopy (Paganin *et al.*, 2002 ▸; Paganin *et al.*, 2004 ▸), where db_*s*_(*x*) = δ_*s*_(*x*)/β_*s*_(*x*) may be *x*-dependent if an *x*-dependent effective spectrum is used. Operationally, this relation is used to express 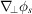 in terms of 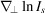 only.

#### 4.3.3. Remark on spatially varying spectra

The implementation supports slow spatial variations of the effective spectrum across the detector (*e.g.* due to filtration, container thickness, or source/optics non-uniformity). If such variations are negligible, one can use a spatially uniform spectrum. Detailed file format and the blockwise interpolation strategy are provided in the supporting information.

### 4.4. Iterative inversion (local and non-local)

#### 4.4.1. Unknowns and single-thickness constraint

In the polychromatic model equation (5), we represent the object-plane transmission as a set of nonnegative spectral components 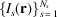 (each *I*_*s*_ already includes its local spectral weight). Under the homogeneous-composition/single-material hypothesis, these components are constrained by a single nominal thickness field. We enforce this by parameterizing 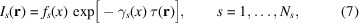
with *f*_*s*_(*x*) the local spectral fraction and γ_*s*_(*x*) the per-line attenuation scaling.

#### 4.4.2. Iteration structure

At iteration *n*, given 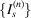, we compute per-line predictions and their sum, form the detector residual, split it across spectral lines, transport the per-line residuals back to the object grid [identity in the local solver, and the corresponding adjoint (transpose) operator equation (4) in the non-local solver], and apply a per-line FFT-diagonal inverse of the linear operator (Paganin-type) as a preconditioner update.

#### 4.4.3. Projection to the single-thickness manifold

After each iteration (for *N*_*s*_ > 1), we project the updated components back to the single-thickness form equation (7). This stabilizes the inversion and prevents drift into non-physical degrees of freedom. Implementation details are given in the supporting information.

## 5. Conclusion

We have presented an accelerated second-order formulation of eikonal phase retrieval for near-field propagation-based imaging, obtained by retaining the complete *O*(*L*^2^) content of a saddle-point (WKB) expansion of the Fresnel propagator. The resulting forward model combines the quadratic eikonal transport contribution (WKB0) with the leading wave-optics correction (diffraction-pressure, WKB1), while remaining expressible through transverse derivatives evaluated in the object plane. This yields an efficient iterative scheme naturally preconditioned by FFT-diagonal single-distance inverse operators.

To remain robust beyond the sub-pixel regime, we complemented the local *O*(*L*^2^) closure with a non-local mapping-based solver that explicitly transports intensity by a mass-conserving forward remapping and transfers detector residuals back to the object grid with the corresponding discrete adjoint (transpose) operator before applying the 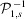 preconditioner. The two solvers therefore form a consistent bridge between regimes where refraction-induced shifts are small and regimes where multi-pixel transport dominates.

The same framework supports a practical polychromatic treatment by discretizing the spectrum into a finite set of effective spectral lines and summing their propagated contributions. In the HiP-CT context, this improves agreement with experimental data by accounting for spectral effects and thereby reducing residual bias and artefacts that cannot be captured by a single effective energy.

Compared with the original EPR implementation reported by Mirone *et al.* (2025*a* ▸), the present approach reduces the phase-retrieval wall time per comparable volume by approximately a factor of 580 when normalized to the same GPU resources (about 2.8 orders of magnitude), making nonlinear phase retrieval compatible with routine, high-throughput reconstruction workflows.

### 5.1. Practical impact and integration in reconstruction workflows

In the current NightRail workflow, phase retrieval and tomographic reconstruction are scheduled to exploit hardware concurrency. When a single GPU is available, phase retrieval can be executed on CPUs while the GPU performs back-projections. For large acquisitions processed in chunks, the workflow overlaps computation: while the GPU back-projects the current chunk, the CPUs pre-process the next chunk, including the phase-retrieval step. When two GPUs are available and the EPR-WKB option is active, one GPU can be reserved for phase retrieval while the other GPU concurrently executes the back-projections. Because the algorithmic complexity of backprojection is higher than that of FFT-based filtering, the additional time required by EPR-WKB can often remain hidden behind tomographic reconstruction in practical high-throughput settings.

Beyond the specific HiP-CT use case, fast access to a second-order near-field model with an explicit wave-optics correction opens the possibility to explore propagation distances closer to the near-field boundary (and potentially slightly beyond it), where standard first-order, linearized single-distance approaches become unreliable. This operating space is particularly relevant for applications pushing towards stronger phase gradients, such as lower-energy imaging and/or higher spatial resolution at modern synchrotron sources, where improved artefact control and quantitative robustness are critical.

## 6. Related literature

The following references, not cited in the main body of the paper, have been cited in the supporting information: Gureyev & Wilkins (1998 ▸); Mandel & Wolf (1995 ▸); Saleh & Teich (1991 ▸).

## Supplementary Material

Supporting information. DOI: 10.1107/S1600577526004273/gy5091sup1.pdf

## Figures and Tables

**Table 1:** Indicative run times for a dataset of 8000 radiographs of size 256 × 3104 pixels All timings include radiograph reading (I/O) and phase retrieval, but exclude tomographic backprojection. CPU timings were obtained on a 96-core server (AMD EPYC 75F3 class), and GPU timings on one NVIDIA A40.

Configuration	*N*_*s*_	Iteration	Time
*L*^1^ mono (Paganin) on CPU	1	1	45 s
Local *L*^2^ mono on GPU	1	1	1.1 min
Local *L*^2^ mono on GPU	1	2	1.23 min
Local *L*^2^ mono on CPU	1	1	1.9 min
Local *L*^2^ mono on CPU	1	2	2.7 min
Local *L*^2^ poly on GPU	5	1	1.47 min
Local *L*^2^ poly on GPU	5	2	1.9 min
Local *L*^2^ poly on CPU	5	1	6.7 min
Local *L*^2^ poly on CPU	5	2	11.3 min
Non-local poly on GPU (oversampling 2)	5	1	2 min
Non-local poly on GPU (oversampling 4)	5	1	8 min
Original EPR implementation	5	Many	870 min

## Data Availability

The code sources to reproduce the shown images and a data subset can be retrieved as detailed in the supporting information.
