# Supplementary material for: Fast eikonal phase retrieval for high-throughput beamlines
Source: J Synchrotron Radiat. 2026 Jun 19;33(Pt 4):1169–79. doi: 10.1107/S1600577526004273 (PMC13344545; doi:10.1107/S1600577526004273)
Supplement: Supplementary file 1 [file s-33-01169-sup1.pdf]

JOURNAL OF SYNCHROTRON RADIATION

Volume 33 (2026)

Supporting information for article:

Fast eikonal phase retrieval for high-throughput beamlines

Alessandro Mirone, Theresa Urban, Joseph Brunet, Claire L. Walsh, Peter D. Lee and Paul Tafforeau

Supporting Information

8.1. Supplementary Methods: Fresnel propagation, WKB expansion, and the diffraction-pressure term

This Supporting Information provides the detailed derivations that underpin the forward models and terminology used in the main text (WKB0/WKB1, local $O(L^2)$ closure, and the diffraction-pressure term). The goal is twofold: (i) to make the mathematical chain of approximations explicit, from the Helmholtz equation to the Fresnel propagator and its WKB approximation, and (ii) to clarify the physical meaning of the WKB1 contribution as the leading wave-optics correction within an otherwise eikonal, point-to-point mapping framework.

8.1.1. From the Helmholtz equation to the Fresnel propagator as a Green's function

Scalar Helmholtz equation and paraxial reduction. We start from the monochromatic scalar Helmholtz equation for a field $U(x, y, z)$ in free space,

$$\left(\nabla_\perp^2 + \partial_{zz} + k^2\right) U(x, y, z) = 0, \tag{S1}$$

where $\nabla_\perp^2 = \partial_{xx} + \partial_{yy}$ is the transverse Laplacian and $k = 2\pi/\lambda$. Introducing the standard slowly varying envelope ψ through

$$U(x, y, z) = e^{ikz}\, \psi(x, y, z), \tag{S2}$$

and substituting into Eq. (S1) yields

$$\nabla_\perp^2 \psi + \partial_{zz}\psi + 2ik\, \partial_z \psi = 0.$$

Under the paraxial approximation (slow longitudinal variation of the envelope), $\partial_{zz}\psi$ is neglected compared with $2ik\, \partial_z\psi$, leading to the paraxial wave equation

$$2ik\, \partial_z \psi + \nabla_\perp^2 \psi = 0. \tag{S3}$$

Equation Eq. (S3) is equivalent to a Schrödinger-type evolution in the propagation variable z,

$$i\,\partial_z\psi = -\frac{1}{2k}\,\nabla_\perp^2\psi. \tag{S4}$$

Green's function and Fresnel integral. The solution of Eq. (S3) with initial condition $\psi(\boldsymbol{r},0)$ can be written using the Green's function (propagator) $G(\boldsymbol{r}'-\boldsymbol{r};L)$ satisfying the same equation and a δ-source at $z=0$:

$$\psi(\boldsymbol{r}',L) = \int_{\mathbb{R}^2} G(\boldsymbol{r}'-\boldsymbol{r};L)\,\psi(\boldsymbol{r},0)\,d^2\boldsymbol{r}.$$

The free-space paraxial Green's function is

$$G(\boldsymbol{\rho};L) = \frac{k}{2\pi i L}\,\exp\!\left(i\frac{k}{2L}\,|\boldsymbol{\rho}|^2\right), \qquad \boldsymbol{\rho} = \boldsymbol{r}'-\boldsymbol{r}, \tag{S5}$$

so that

$$\psi(\boldsymbol{r}',L) = \frac{k}{2\pi i L}\int_{\mathbb{R}^2}\psi(\boldsymbol{r},0)\,\exp\!\left(i\frac{k}{2L}\,|\boldsymbol{r}'-\boldsymbol{r}|^2\right)\,d^2\boldsymbol{r}. \tag{S6}$$

Recovering $U = e^{ikz}\psi$ gives the standard Fresnel (paraxial Huygens–Fresnel) integral

$$U(\boldsymbol{r}',L) = \frac{k}{2\pi i L}\,e^{ikL}\int_{\mathbb{R}^2}U(\boldsymbol{r},0)\,\exp\!\left(i\frac{k}{2L}\,|\boldsymbol{r}'-\boldsymbol{r}|^2\right)\,d^2\boldsymbol{r}, \tag{S7}$$

which is therefore the Green's function solution of the paraxial reduction of the Helmholtz equation. This observation is useful because it allows the WKB corrections to be interpreted both from the stationary-phase expansion of Eq. (S7) and from the hydrodynamic (Madelung) form of the paraxial PDE.

8.1.2. Saddle-point (WKB) expansion of the Fresnel integral We work with transverse coordinates $\boldsymbol{r} = (x,y)$ in the object plane ($z=0$) and $\boldsymbol{r}' = (x',y')$ in the detector plane ($z=L$). We write the object-plane field as $U(\boldsymbol{r},0) = A(\boldsymbol{r})\,e^{i\phi(\boldsymbol{r})}$, with intensity $I(\boldsymbol{r},0) = |U(\boldsymbol{r},0)|^2 = A^2(\boldsymbol{r})$. For bookkeeping we introduce

$$\varepsilon \equiv \frac{L}{k} = \frac{\lambda L}{2\pi}, \tag{S8}$$

which has units of length^2. The near-field condition corresponds to a large Fresnel number $N_F = \ell^2/(\lambda L) \gg 1$, where ℓ is a characteristic transverse length scale of variation of A and ϕ (Saleh & Teich, 1991).

WKB0 saddle: eikonal ray map. To put Eq. (S7) into stationary-phase form (Mandel & Wolf, 1995; Gureyev & Wilkins, 1998), we collect the free-space quadratic phase and the object phase into the phase function

$$\Phi(\boldsymbol{r};\boldsymbol{r}') \equiv \frac{1}{2}\,|\boldsymbol{r}'-\boldsymbol{r}|^2 + \varepsilon\,\phi(\boldsymbol{r}), \qquad i\phi + i\frac{k}{2L}|\boldsymbol{r}'-\boldsymbol{r}|^2 = \frac{i}{\varepsilon}\,\Phi. \tag{S9}$$

Stationary points $\boldsymbol{r}_s$ satisfy $\partial_i\Phi(\boldsymbol{r}_s;\boldsymbol{r}') = 0$, giving the near-identity eikonal map

$$\boldsymbol{r}' = \boldsymbol{r}_s + \varepsilon\,\nabla_\perp\phi(\boldsymbol{r}_s). \tag{S10}$$

In the near-field regime $\varepsilon\nabla_\perp\phi$ is small so the mapping remains single-valued (no caustics) and the saddle lies close to $\boldsymbol{r}_s \simeq \boldsymbol{r}'$.

Expanding Φ about the saddle by writing $\boldsymbol{r} = \boldsymbol{r}_s + \boldsymbol{u}$ yields

$$\Phi(\boldsymbol{r}_s+\boldsymbol{u};\boldsymbol{r}') \simeq \Phi_s + \frac{1}{2}\,u_i H_{ij} u_j, \qquad H_{ij} = \delta_{ij} + \varepsilon(\partial_i\partial_j\phi)_s, \tag{S11}$$

with $\Phi_s = \Phi(\boldsymbol{r}_s;\boldsymbol{r}')$. Keeping the amplitude fixed at $A_s = A(\boldsymbol{r}_s)$ and evaluating the Gaussian integral yields the leading WKB0 field

$$U_{\mathrm{WKB0}}(\boldsymbol{r}',L) = e^{ikL}\,\frac{A_s}{\sqrt{\det H}}\,\exp\!\left(\frac{i}{\varepsilon}\,\Phi_s\right). \tag{S12}$$

WKB1 saddle: amplitude curvature and the "diffraction-pressure" phase. The first correction arises by retaining the local curvature of $A = \sqrt{I}$ around the saddle,

$$A(\boldsymbol{r}_s+\boldsymbol{u}) = A_s + (\partial_i A)_s\,u_i + \frac{1}{2}(\partial_i\partial_j A)_s\,u_i u_j + O(|\boldsymbol{u}|^3). \tag{S13}$$

The linear term integrates to zero by symmetry, and the quadratic term yields

$$U(\boldsymbol{r}',L) \simeq U_{\mathrm{WKB0}}(\boldsymbol{r}',L)\left[1 + \frac{i\varepsilon}{2}\,\frac{(\partial_i\partial_j A)_s(H^{-1})_{ij}}{A_s}\right]. \tag{S14}$$

In the near-field regime $H^{-1} = \mathbf{I}_2 + O(\varepsilon)$, so the correction reduces to the local curvature ratio

$$Q(\boldsymbol{r}) \equiv \frac{\nabla_\perp^2 A(\boldsymbol{r})}{A(\boldsymbol{r})} = \frac{\nabla_\perp^2 \sqrt{I(\boldsymbol{r},0)}}{\sqrt{I(\boldsymbol{r},0)}}. \tag{S15}$$

This term is the local diffraction-pressure (or quantum-potential) field. It acts as a local phase correction to the field. Its leading impact on the propagated intensity appears at order $O(\varepsilon^2)$.

WKB0 forward model and local intensity expansion. Neglecting multi-branch interference (single relevant saddle), WKB0 transport can be written as a mass-conserving mapping of intensity along the ray map Eq. (S10):

$$I(\boldsymbol{r}', L) = \int I(\boldsymbol{r}, 0)\, \delta\big(\boldsymbol{r}' - \boldsymbol{r} - \varepsilon \nabla_\perp \phi(\boldsymbol{r})\big)\; d^2\boldsymbol{r}. \tag{S16}$$

Expanding the delta distribution in ε and integrating by parts gives the WKB0 (eikonal) series

$$I(\boldsymbol{r}', L) \simeq I(\boldsymbol{r}', 0) - \varepsilon\, \partial_i\big(I\, \partial_i \phi\big)\Big|_{\boldsymbol{r}'} + \frac{\varepsilon^2}{2}\, \partial_i \partial_j\big(I\, \partial_i \phi\, \partial_j \phi\big)\Big|_{\boldsymbol{r}'} + O(\varepsilon^3). \tag{S17}$$

8.1.3. Diffraction-pressure term from the Madelung form of the paraxial equation

Hydrodynamic (Madelung) representation. Because Fresnel propagation is the Green's function solution of the paraxial equation Eq. (S3), one can equivalently derive WKB corrections by rewriting Eq. (S4) in amplitude-phase variables, i.e. in its hydrodynamic (Madelung) form (Madelung, 1927). Let

$$\psi(\boldsymbol{r}, z) = \sqrt{I(\boldsymbol{r}, z)}\, e^{i\phi(\boldsymbol{r}, z)}.$$

Substituting into Eq. (S4) and separating real and imaginary parts yields the exact pair

$$\partial_z I + \frac{1}{k}\, \nabla_\perp \cdot (I \nabla_\perp \phi) = 0, \tag{S18}$$

$$\partial_z \phi + \frac{1}{2k}\,|\nabla_\perp \phi|^2 - \frac{1}{2k}\,Q = 0, \tag{S19}$$

with Q defined in Eq. (S15). The Q-term is the well-known quantum-potential (quantum-pressure) contribution in the Madelung formulation (Madelung, 1927), and in the present context it is naturally interpreted as a local "diffraction-pressure" correction to purely geometrical transport.

Intensity expansion to $O(\varepsilon^2)$ (WKB0+WKB1). Taylor-expanding

$$I(\boldsymbol{r}', L) = I(\boldsymbol{r}', 0) + L\,\partial_z I|_0 + \frac{L^2}{2}\,\partial_z^2 I|_0 + O(L^3),$$

and using Eq. (S18)–Eq. (S19) to eliminate $\partial_z I$ and $\partial_z \phi$ yields an additional $O(\varepsilon^2)$ contribution to the intensity series, namely $-(\varepsilon^2/4)\,\partial_i(I\,\partial_i Q)$. Combining this with the WKB0 transport terms gives the local WKB0+WKB1 intensity expansion used in the main text:

$$\begin{aligned} I(\boldsymbol{r}', L) \simeq I(\boldsymbol{r}', 0) &- \varepsilon\,\partial_i(I\,\partial_i \phi)\Big|_{\boldsymbol{r}'} \\ &+ \frac{\varepsilon^2}{2}\,\partial_i\partial_j(I\,\partial_i\phi\,\partial_j\phi)\Big|_{\boldsymbol{r}'} - \frac{\varepsilon^2}{4}\,\partial_i(I\,\partial_i Q)\Big|_{\boldsymbol{r}'} + O(\varepsilon^3). \end{aligned} \tag{S20}$$

This derivation emphasizes that the diffraction-pressure term is not an additional model assumption: it is an exact component of the paraxial wave equation expressed in amplitude-phase variables, and it enters the local near-field intensity expansion at the same order as the quadratic eikonal transport correction.

Scope and interpretation of WKB1. In our formulation, WKB0 corresponds to a point-to-point transport map (ray mapping), while WKB1 provides the leading local wave-optics correction compatible with this mapping picture. As one moves further away from the near-field regime, increasingly important physics involves wavefront structure and interference that cannot be fully represented within a single-valued, local mapping framework. This motivates retaining WKB0 and including only the

leading WKB1 term at $O(L^2)$ as a compact estimate of wave-optics effects while staying within the eikonal-WKB setting.

8.2. Supplementary Note: spatially varying effective spectrum (example and general applicability)

This note provides additional context on the x-dependent effective spectrum parameterization used in the polychromatic implementation. The same formalism applies to other beamlines and configurations whenever the transmitted spectrum varies across the field of view (e.g. due to filtration, sample container thickness, or source/optics non-uniformity). If such variations are negligible, the model reduces to a spatially uniform spectrum (single block).

Why an x-dependent spectrum can arise. In some PPC-μCT configurations, specimens are immersed in a cylindrical container whose axis is aligned with the vertical detector direction. In such cases, upstream filtration introduced by the container (and by the specimen itself) varies predominantly across the horizontal coordinate x: rays crossing the jar center traverse a longer path length in the container material than rays closer to the lateral sides. For sufficiently large containers, this alone can produce a marked spatial variation of the transmitted effective spectrum (beam hardening stronger at the center and weaker towards the edges).

Example: BM18 HiP-CT. On BM18, the incident spectrum can also be non-uniform across x because of the tripole wiggler design: the final beam results from the superimposition of emission from a central pole (higher field) and two lateral poles (lower field). In the HiP-CT operating regime (around the equilibrium energy, about 100 keV), these effects combine in a non-trivial way, motivating an x-dependent effective spectrum parameterization.

Extensions. A dependence on the vertical coordinate y is straightforward to include if required (e.g. by tabulating two-dimensional spectral maps instead of one-dimensional profiles). In many helical acquisition settings, a purely x-dependent model is already a robust compromise because a detector row is not associated with a unique physical height throughout the scan.

8.3. Data availability and reproducibility (NightRail)

8.3.1. Demo dataset and NightRail input files All examples shown in this paper (including the demo dataset and the corresponding NightRail input configuration files) are available at:

`https://doi.org/10.5281/zenodo.19445796`

The archive contains a ready-to-run `RECONSTRUCTIONS` directory with multiple reconstruction cases matching the configurations discussed in the manuscript.

8.3.2. Installing the NightRail BM18 workflow and running the examples In addition to the demo archive above, reproducing the results requires installing the NightRail BM18 application code. The installation is performed in three steps.

Step 1: clone the repository. Clone the repository and check out the `eprwkb` branch:

```
git clone https://gitlab.esrf.fr/night_rail/applications/mirone/night_rail_bm18
cd night_rail_bm18
git checkout eprwkb
```

Step 2: create the Python virtual environment and install the code. From the cloned directory, enter `install_u24_venv` where you find the installation script.

Step 3: adapt the installation script. Edit the script

```
no_surprise_installation_script_u24.sh
```

to select the desired target directory for the virtual environment. In particular, set:

```
TAG=eprwkb
```

and run the script to complete the installation.

8.3.3. Running an example reconstruction All demo cases are prepared ready to run without modification. As a representative example, consider the directory `RECONSTRUCTIONS/epr_mono_3iter`. From the demo directory:

```
cd RECONSTRUCTIONS/epr_mono_3iter
```

Generate the workflow scripts (graph expansion).

```
night_rail_bm18_create_scripts -i nrbm18_default.json
```

Run the workflow scripts. Then execute the generated session script:

```
./unnamed_session_run_graph.sh
```
